# The role behind the scenes of Tregs and Th17s in Hashimoto’s thyroiditis: Toward a pivotal role of FOXP3 and BACH2

**DOI:** 10.3389/fimmu.2022.1098243

**Published:** 2022-12-12

**Authors:** Alessio Mazzieri, Pia Montanucci, Giuseppe Basta, Riccardo Calafiore

**Affiliations:** ^1^ Translational Medicine and Surgery, Department of Medicine and Surgery, University of Perugia, Perugia, Italy; ^2^ Division of Internal Medicine and Endocrine and Metabolic Sciences (MISEM), Laboratory for Endocrine Cell Transplants and Biohybrid Organs, Department of Medicine and Surgery, University of Perugia, Perugia, Italy

**Keywords:** Hashimoto’s thyroiditis, autoimmunity, imbalance Th17/Treg, *BACH2*, *FOXP3*

## Abstract

In Hashimoto’s thyroiditis (HT), the genetic bases play a central role in determining development of the disease. In particular, the most frequent genes involved in the onset of HT are the Human Leukocyte Antigen (*HLA*). However, there are other genes and transcription factors in the autoimmune background of HT, both isolated and as part of autoimmune polyendocrine syndromes (APS). Recently more interest is being fueled toward *BACH2* (BTB Domain and CNC Homolog 2), that promotes Tregs (T regulators lymphocytes) differentiation and enhances Treg-mediated immunity. The synergistic interaction between environmental agents and the aforementioned genes leads to the onset of autoimmunity and ultimately to damage of the thyroid gland. In this scenario, the role of Th17 (T helper-17 lymphocytes) and Treg cells is still less defined as compared to action of Th1 cells (T helper-1 lymphocytes) and cytotoxic lymphocytes (CD8 ^+^ T lymphocytes). Evidences show that an imbalance of Th17/Treg ratio represents a prognostic factor with respect to the gland damage. Moreover, the deficient ability of Treg to inhibit the proliferation of T cells against the self can break the immune balance. In light of these considerations, the use of genetic panels and the progress of immunotherapy could allow for better targeting treatment and preventive interventions in subjects with potential or early stage of HT.

## 1 Introduction

In Hashimoto’s thyroiditis (HT), the genetic background plays a fundamental role in inducing development of its serological hallmarks: TPOAb (Anti-Thyroperoxidase antibodies) and/or TgAb (Anti-Thyroglobulin antibodies). In the set of the genetic factors involved in HT, thyroid-specific genes such as TSHR (TSH receptor) and Thyroglobulin (Tg) are to be highlighted (1). Other genes, involved in antigen presentation, thereby implicated in the onset of HT are the Human Leukocyte Antigen (*HLA*), immunoregulatory genes. Moreover, derangements of peripheral tolerance can participate in the development of the disease. Mutations of *FOXP3* (Forkhead box P3), a gene regulated by *BACH2* (BTB Domain and CNC Homolog 2), have in fact been associated with increased prevalence of HT in Caucasian populations (2), especially in women (due to the location of *FOXP3* on X chromosome). Another element in favor of the female prevalence of HT could also lie in self-antigens expression in X chromosome, inactivated by lyonization; perhaps the deficit in their expression can affect the tolerogenic properties of the immune system (3).

Another gene involved in central tolerance is *AIRE* (Autoimmune Regulator Gene); its loss of function can lead to the APS1 (autoimmune polyendocrine syndromes type 1). Nevertheless, alterations of the *AIRE* represent only a small percentage of HT (0.3-0.6%) (4).

Like genetic factors, environmental factors also play, albeit to a lesser extent, a role in the onset of HT. A correlation between the intake of iodine (5) (useful for prevention of nodular thyroid disorders) and the increased risk of HT is documented, without a clear explanation.

Other possibly involved environmental factors, are Selenium (6) (due to the anti-oxidizing action of selenoprotein glutathione-peroxidase), Myo-inositol and vitamin D (7, 8), with weaker evidence. Moreover, also voluptuous habits (as smoke) can participate at the onset of HT or otherwise.

In the cohort studies by Strieder (9) et al. and Belin (10) et al. a lower level of TPOAb was observed in smokers than in non-smokers. The mechanism by which tobacco may reduce the risk for HT is not yet fully understood. As it happens for other endocrine glands, infections can also be a precipitating factor in the immune background with the consequent development of autoimmunity.

Hepatitis C Virus, Parvovirus B19 and some enteroviruses (11), usually through molecular mimicry mechanisms, are the most likely infectious agents associated with the onset of HT.

## 2 Autoimmune bases

The pathogenic development of HT is still cloudy, due to the variety of risk factors involved. At the moment, further studies are needed to evaluate the synergistic interaction between environmental agents and genes for the onset of this disease.

In a recent review, Weetman compares the pathogenesis of HT to a “Swiss cheese” model: the cumulative effects of defects line up, like holes in cheese slices, to induce the autoimmune destruction pathways (12).

When the immune regulation against the self fails, autoimmunity will ensue. In the specific case of HT, damage mechanisms strike the thyroid gland. The target antigens of the immune system, as above mentioned, are TPO and Tg.

When these antigens are exposed to recognition of the immune system, an inflammatory response leads to an invasion of the thyroid gland with a massive predominantly B lymphocyte infiltrate (up to 50%), then CD8^+^ and CD4 ^+^ T lymphocytes (13).

Different populations of T lymphocytes play different roles in the chain of pathogenic events. Th1 (T helper-1 lymphocytes) cells act by stimulating cytotoxic lymphocytes and macrophages for direct damage to the follicle, throughout its destruction. Moreover, Th1s induce plasma cells to produce antibodies directed against the antigens of the gland (Ab anti-TPO, Ab anti-TG), that are actually humoral markers of the disease rather than pathogenic elements. CD8 ^+^ T lymphocytes act by a direct cytotoxic action, instead. The role of Th17s (T helper-17 lymphocytes) and Tregs (T regulators lymphocytes) in this “game” is still less defined. In patients with HT, the peripheral Treg count is variable in individual cases; the comparison with count of healthy subjects may show decreased, equal or even increased numbers. However in HT, part of the immune imbalance lies in the deficient ability of Tregs to inhibit the proliferation of T cells against the self (14). The humoral finding of the lack of regulation comes from the elevated levels of IL-17 (Interleukin-17) and IL-22 (Interleukin-22) in the blood like in the thyroid tissue (15). On the other hand IL-10 (Interleukin-10), secreted by Treg, regulates the action of Th17s and plays a protective role against the damage of the thyroid gland. In fact, several evidences show that the Th17/Treg imbalance represents a prognostic factor with respect to the damage suffered by the gland, with a direct relationship with TPOAb, TgAb (14).

Another peculiar aspect of patients with HT is the decreased concentration in peripheral blood of the plasmacytoid Dendritic Cells (a subtype of activated Dendritic Cells and usually involved in the viral response) at the expense of a normal concentration of conventional immunogenic dendritic cells (16). This could be significant due to the fact that Dendritic Cells play an active role in tolerance: presentation of surface receptors such as P-Selectin glycosylated ligand-1 (PSGL-1), Ligands of the programmed death 1 receptor (PD-L1, PD-L2); expression of indoleamine 2,3-dioxygenase (IDO) (17), that generates metabolites of tryptophan actively participating in Treg differentiation.

## 3 Genetic factors

In addition to the well-known *HLA*, other genes and transcription factors are involved in the pathogenic mechanisms of HT. Recently particular interest has been turned on *FOXP3* and *BACH2*.

In future, extensive genomes scans may be needed to determine the possible further genetic contribution to the development of HT.

### 3.1 HLA

The *HLA* complex (18) refers to a group of genes of 700 kilobases located on chromosome 6p21.3 encoding the proteins presenting the antigen, on the cell surface.

Certains *HLA* show a greater propensity to develop autoimmune disorders (e.g. *DR/DQ* allelic combination); others exhibit a protective mechanism for the self. In the case of HT, the polymorphism of the *DQ* locus is frequently involved. Each individual with *HLA-DQ* isoform can present different antigens to T cells, which can be mature and stimulate the production of antibodies by B cells. However, when the tolerance breaks up, the autoimmune process can start. In fact, there is strong evidence on how the *DQ* alleles are the most important susceptibility markers for the development of autoimmune diseases through their ability to modify the configuration of the antigenic binding site. HLA-A * 02: 07 and *HLA-DRB4* have been associated with susceptibility to Hashimoto’s thyroiditis, while HLA-A * 33: 03-C * 14: 03-B * 44: 03-DRB1 * 13: 02 -DQB1 * 06: 04-DPB1 * 04: 01 were identified as protective genes. Specifically, APS2 is associated with HLA-DQB1 * 0201 and DQB1 * 0302 or with haplotype HLA-DRB1 * 03-DQA1 * 0501-DQB1 * 0201.

### 3.2 FOXP3

*FOXP3* has provided relevant information on the generation and maintenance of Tregs. Its transcription is induced by T cell receptor signalling (TCR); in particular, temporally persistent TCR signals activate *FOXP3* transcription in autoreactive thymocytes (19). Upon its expression, a self-regulating transcriptional circuit stabilizes the expression of *FOXP3* to consolidate the differentiation of Tregs and activate the suppressive function. However, these molecular mechanisms are not yet fully understood. The analysis about the genetic expression of FOXP3 ^+^ T and FOXP3^-^ T cells also suggested that many specific genes of Tregs are independent of *FOXP3* (20). These results change the paradigm of *FOXP3* as the only factor required for the development of Treg cell phenotype.

**Figure 1:**
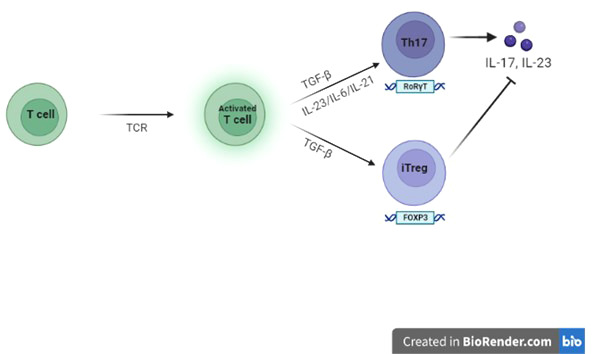
Role of FOXP3 on Tregs and Th17s In the context of an adequate pro-inflammatory microenvironment for specific Interleukins, TGF-β, the cytokine-guide for the differentiation of iTregs (induced Tregs), promote the differentiation into Th17s.

However, determination of Treg cell fate may be influenced by *FOXP3* interaction with elements related to TCR-mediated T cell activation, such as IL-2 (Interleukin-2) and TGF-β (Transforming growth factor-β) signalling pathways (21). TGF-β induces *FOXP3* and by the binding to its receptor I (TGF-βRI), stimulates the differentiation of Tregs. Experts also suggest a possible anti-apoptotic role for TGF-β that would improve the survival of nTregs (natural Tregs) and thus contribute to their stability (22). In the study by B. Kristensen et al. (23), it was shown that IL-6 (Intereukin-6) and TGF-β are important for human Th17 differentiation; perhaps, patients with HT have a higher basal production of IL-6 and TGF-β1 than healthy donors. Indeed, Tregs can also be reprogrammed into Th17s *via* IL-6- and IL-1β-dependent signalling. This process is mediated by the activation of STAT3 (Signal transducer and activator of transcription 3), RORγt (Retinoic acid-related orphan receptor gamma t) and RORα (Retinoic acid-related Orphan Receptor alfa) which downregulate the expression of *FOXP3* and promote the conversion of Tregs into Th17s (**Figure 1**). Actually, in Kristensen’s study, the basal expression of FOXP3 mRNA show an attenuation in patients with HT, but this expression is quantitatively very similar between patients and healthy controls.

In pathogenesis of autoimmunity, the concept of an insufficiency of CD4^+^FOXP3^+^ Tregs is opposed by a theory postulating their functional deficit, rather than a numerical defect of them. In fact in HT, as well as in other autoimmune diseases, the self-reactive T cells would be resistant to suppression, not so much due to the low number of Tregs, but due to their regulatory inability. In support to this theory, a gene splicing variant *FOXP3Δ2*, devoid of exon 2, unable to inhibit Th17 differentiation, compared to the full-length form of *FOXP3* has been documented. Indeed, in Kristensen’s study, the baseline expression of the mRNA encoding *FOXP3* is similar in HT patients and healthy donors; however, HT patients show a higher constitutive expression of the *FOXP3Δ2* splice variant (59-67% % vs approximately 30%) (23). Therefore, the increase of IL-6 and TGF-β1 in the microenvironment and the increased expression of *FOXP3Δ2* may contribute to the shift towards Th17s in patients with HT.

Full-length *FOXP3* is known to suppress RORγT, RORα, activated T cell nuclear factor (NFAT) and nuclear factor-kappa B (NF-κB) signalling. Conversely, FOXP3Δ2 is incapable of these trophic effects (23). Due to RORγt, the essential transcription factor for Th17s and RORα, highly expressed in Th17s (induced by TGF-β/IL-6), the relative overexpression of *FOXP3Δ2* in persons with HT may compromise the inhibition of Th17 differentiation. On the other hand, the interaction of *FOXP3Δ2* with GATA3 (GATA binding protein 3), can cause a reciprocal increase in its expression; this interaction is also involved in the control of Th2s (T-helper 2 lymphocytes) responses by Tregs. Instead, in the environmental presence of IFN-γ, Tregs promote their own expression of T-bet (T-box transcription factor, pivotal to the development of Th1s) (24). Conversely, this factor induces the upregulation of CXCR3 (C-X-C Motif Chemokine Receptor 3), that allow Tregs to migrate into inflamed tissues and inhibit Th1 responses (25). In this regard, in a study by Tokić et al. (26), a similar upregulation of *FOXP3* and T-bet was shown in HT patients compared to healthy subjects. Specifically, there was also an increased expression in hypothyroid as compared to euthyroid HT patients. Actually, these data are correlated with the stage of disease, rather than the thyroid hormone levels.

In violation of immunological tolerance, changes in microenvironmental signals disrupt the transcriptional and epigenetic regulation of *FOXP3* (19), resulting in reduced Treg cell generation and suppressive function. In fact, the activity of FOXP3 is influenced by possible post-translational modifications such as acetylation. Acetylation of specific *FOXP3* lysine residues increases the stability of *FOXP3* and its ability to bind DNA and activate specific effector functions. A key molecule involved in the acetylation process is TAZ (transcriptional coactivator PDZ-binding) which plays a vital role in driving Th17 cell differentiation and inhibiting Treg cell development. Indeed, TAZ is a co-activator of RORγT and limits the differentiation of Tregs by decreasing *FOXP3* acetylation. Impaired *FOXP3* acetylation has been associated with the pathogenesis of several autoimmune diseases, such as HT.

These results indicate that the regulation of *FOXP3* and the subsequent function of Tregs are based on different molecular processes; pharmacological manipulation of these pathways could pave the way for novel immunological tools to control Treg cell function and immunological tolerance in immune-related disorders.

The major disorder associated with loss of Treg cell function is an immune-dysregulation syndrome, poly-endocrinopathies, X-linked enteropathy (IPEX) (27), characterized by germline mutations of *FOXP3*, in both non-coding and coding sequences. Thus, IPEX subjects with mutations express a functionally defective FOXP3 protein, resulting in reduced suppressive activity of Tregs. These findings underscore the importance of *FOXP3* in controlling the function of Treg cell and how its related defects contribute to pathogenesis and progression of autoimmunity.

### 3.3 BACH2

*BACH2* (located on chromosome 6q15) is a gene recently studied in the pathogenesis of several autoimmune diseases. Historically known in monocytes and neurons, further studies have shown its expression in B and T cells (28). The exact molecular mechanisms by which *BACH2* protects hosts from the development of autoimmunity are not completely yet understood.

**Figure 2:**
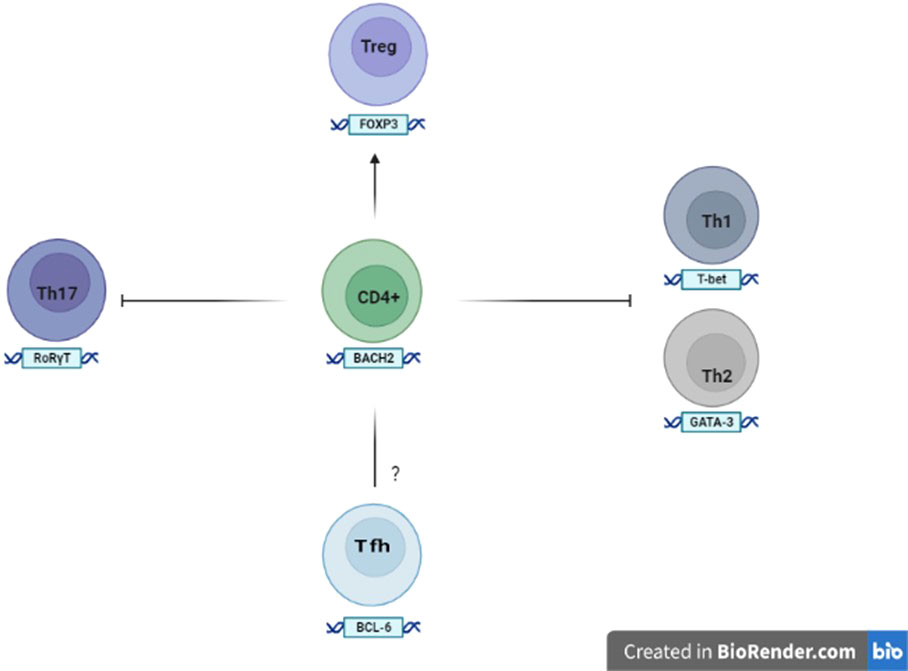
Expression and role of BACH2 in the T cells The expression of BACH2 promotes the differentiation of Tregs, while at the same time suppresses the factors involved in the formation of the Th1, Th2 and Th17 subsets. The role of BACH2 in the development and differentiation of Tfh (follicular T helper lymphocytes) remains to be elucidated.

*BACH2* works within innate and adaptive lines to control immune responses. It is highly expressed in B cells of the germinal centers (GCs), where it promotes the change of antibody class and suppresses the differentiation of plasmacells. *BACH2* is also expressed by T cells and directs T-helper cell differentiation, homeostasis and effector functions (29). In fact, it limits the complete differentiation of Th1, Th2 and Th17 cells and generally keeps Th cells in a naive state by suppressing memory-related effector genes (**Figure 2**). *BACH2* also promotes FOXP3 ^+^ regulatory T cell differentiation and enhances Treg-mediated immunity. Moreover, *BACH2* suppresses the differentiation of Th2s, through the down-regulation of GATA-3, the main transcription factor of this cell subtype.

Some studies also show that the ablation of *BACH2* on T cells is associated with the expansion of follicular helper T cells (Tfhs) and abnormal GCs with consequent humoral autoimmunity (29). Tfhs are generally considered to be a separate subset of Th cells and derived from naïve CD4 ^+^ T cells after sequential steps in response to certain antigens. Naive CD4 ^+^ T cells triggered by dendritic cells upregulate the chemokine receptor CXCR5 (C-X-C chemokine receptor type 5), which allows them to migrate into B-cell follicles to further differentiate into Tfhs. However, Tfhs can also be generated by the conversion of other effector T cells under conditions of chronic and sustained antigenic stimulation. The differentiation of Tfhs is controlled by specific molecules, including the transcription factor c-Maf (Musculoaponeurotic fibrosarcoma), CXCR5 (suppressed by *BACH2*) and BCL6 (B-cell lymphoma 6) that downregulates *BACH2*. Tfhs, with loss of *BACH2* in differentiation, are diverted to a subset cell that produces IL-4 (Interleukin-4), inducing switching of the IgG1 (Immunoglobuline Gamma G1) and IgE (Immunoglobuline E) histotypes of B cells.

Within GCs, Tfhs interact with B cells *via* co-stimulatory and co-inhibitory molecules, including programmed cell death protein 1 (PD-1), to facilitate selection and maturation of B cells with high affinity. However, excessive Tfhs impair positive selection by reducing competition between B cells and thus resulting in a lower threshold for selection which allows for the emergence of autoreactive clones (29).

Genetic polymorphisms of *BACH2* are associated with numerous autoimmune and allergic diseases in humans.

Deficient *BACH2* mice exhibit Treg cell depletion, decreased *FOXP3* expression, and at the same time increased Th2-related cytokine production, leading to the development of fatal inflammatory lung disease (30). In humans, *BACH2* haplo-insufficiency results in a syndrome with defective lymphocyte maturation that causes immunodeficiency, recurrent broncho-pulmonary infections and intestinal inflammatory processes (31).

Polymorphisms of *BACH2* have been found to be associated with several autoimmune conditions, including vitiligo, rheumatoid arthritis and systemic lupus erythematosus; in particular, the rs3757247 variant has been associated with APS. For this reason, even with regard to HT, the main studies are currently aimed at cases of polyglandular autoimmunity (28). A Polish population study clearly demonstrated an association of *BACH2* polymorphism rs3757247 with APS (31). These results and other limited studies indicate *BACH2* as one of the universal genetic markers of autoimmunity. However, establishing a reliable set of polymorphisms to predict a possible multiple autoimmunity is a hypothetical subject of further analysis.

The search for variants of *BACH2* could be a part of the early diagnosis panel that identifies individuals prone to develope a polyglandular autoimmunity, including those already diagnosed with an autoimmune disorder or in first-degree relatives of patients with APS.

## 4 Conclusions

Although huge strides have been made with regard to our knowledge on the causes of HT, the genetic and non-genetic factors involved in its pathogenesis are more complex than could be yet imagined. Additional work in this area is needed to confirm the molecular associations, identify any causal relationship and explore the immunological implications.

Recent studies show how in the pathogenic history of HT to the classic role of Th1s escaped from tolerance mechanisms, should be added the counterpart, consisting of self-reactive Th17s and reduced or ineffective Tregs.

Kristensen’s study (23) showed greater expression of TGFβ1 messenger in thyroiditis as compared to controls. The data described, known the double role in differentiation of the cytokine-guide TGFβ1 and the systemic inflammatory environment in HT, could explain the lymphocytic switch towards Th17s. About *FOXP3* in Kristensen’s study, its expression was substantially similar in patients and healthy subjects. On the other hand, in the study conducted by Tokić (26), the expression of *FOXP3* messenger was higher in patients than in controls. This finding could be related to a functional defect of Tregs, present in patients with HT but not in controls.

At this juncture, HT is considered the archetype for autoimmune disease; due to the global spread of HT, subjects with this disease represent an ideal model for genetic and immunologic studies. In fact, in the last two decades, there has been huge interest in unravelling its genetic factors and autoimmune bases. In future, the use of genetic panels and the progress of immunotherapy could allow targeted treatment and preventive interventions in autoimmune endocrine diseases. A reliable identification of subjects at high risk of autoimmunity will be crucial for selecting the appropriate candidates for new treatments. Possible immunotherapeutic approaches coordinated with genomic screening, for HT as well as for other autoimmune diseases, should not be seen as a replacement to current gold-standard therapies (levothyroxine monotherapy as the standard of care; levothyroxine/liothyronine combination, in patients with persistent symptoms of hypothyroidism) (32). Nevertheless, the progress in genetic field and in immunotherapy can be seen as a chance to solve the immunological imbalance in high-risk persons and in patients with early stage of the disease or subsequently.

## Author contributions

AM wrote the manuscript. PM and GB contributed to the redaction of the review. RC contributed as senior author to the supervision of the final manuscript. All authors contributed to the article and approved the submitted version.
